# Exploring the Association between Emotional Intelligence and Academic Performance and Stress Factors among Dental Students: A Scoping Review

**DOI:** 10.3390/dj10040067

**Published:** 2022-04-07

**Authors:** Shah Saif Jahan, Jayashri Tamanna Nerali, Ali Davod Parsa, Russell Kabir

**Affiliations:** 1 Faculty of Health, Education, Medicine and Social Care, School of Allied Health, Anglia Ruskin University, Chelmsford CM1 1SQ, UK; ssj151@student.aru.ac.uk (S.S.J.); ali.parsa@aru.ac.uk (A.D.P.); 2 General Dentistry, Penang International Dental College, Butterworth 12000, Malaysia; jnerali@pidc.edu.my

**Keywords:** scoping review, emotional intelligence, dental students, academic performance, stress

## Abstract

Background: Numerous studies have been conducted to explicate the scope of emotional intelligence in educational success and coping with stress in different academic sectors, but very few have been conducted with dental students. This scoping review aimed to ascertain the role of emotional intelligence in academic performance and stress factors among dental students. Methods: All publications in the English language between 2001 and 2020 were retrieved employing MeSh keywords. Academic resources such as Pubmed, Pubmed Central, EMBASE, Web of Science, EBSCO-Host, Cochrane, PROSPERO, and ARU E-library were comprehensively searched for empirical research. One thousand, three hundred and fifty-nine papers were screened according to the Preferred Reporting Items for Systematic Reviews and Meta-Analyses (PRISMA) standards for inclusion and exclusion criteria. These publications were then evaluated further by deleting duplicates, examining full-text articles, and conducting an abstract assessment. This review included a critical appraisal of 24 articles. Results: The narrative analysis method was applied to evaluate the data retrieved from publications regarding EI, academic performance, and stress factors. The review found that EI had a greater impact on the educational success of dental students throughout their clinical years. Moreover, EI may be a key tool in coping with stress and negative emotions. Higher EI scores were shown to be associated with better performance in organizational and leadership abilities, which are important for career advancement. Conclusion: The review suggested including EI training in the dental curriculum. Furthermore, EI should be used as a selection criterion for admission to dental education.

## 1. Introduction

Emotional intelligence is defined as a person’s capacity to comprehend and recognize their own and others’ emotions, use cognitive awareness to regulate actions and behavior, and adjust their feelings to challenging circumstances [1]. Additionally, emotional intelligence exhibits the potential of intellect, perception, and sense to enhance the reasoning and understanding of interpersonal complexities [2]. It is considered that individuals with a high EI score have a more heightened sense of self-worth, superior employment status, and management ability. A high EI suggests the increased capacity for comprehending, controlling, and managing emotional stress [3]. Students with a high degree of emotional intelligence (EI) may be more efficient at individual and academic levels as they can regulate their emotions better than others [4]. Individuals who are emotionally intelligent are not only conscious of their own feelings, but they also express compassion and an understanding of the sentiments of others around them [5]. It was previously assumed that emotional intelligence is an inherent and quantifiable talent that cannot be modified [6], but later it was found that EI abilities can be imparted, and an individual’s EI can be developed [7,8]. Higher education may be an excellent place for teaching emotional intelligence abilities [9]. No significant amount of study has been carried out on the function that emotional intelligence plays in the education of undergraduates in the health professions, and most of the research that has been carried out is in nursing and medical education [10,11,12]. Despite evidence of the relevance of clinical faculty members having high emotional intelligence abilities [13,14], there is a scarcity of research into the function that emotional intelligence has in the dental education context.

Interpersonal and intrapersonal intelligence are components of EI. Interpersonal awareness enables people to perceive and establish good interaction with one another. While intrapersonal intelligence is a synthesis of the self-awareness, self-control, and drive that are necessary for self-evaluation and recognition [15], the ability to communicate effectively, empathize, and build harmonious relationships with others is a sign of interpersonal intelligence. They can rapidly perceive the temperament, nature, and personality of others, as well as their emotions, goals, and intents. These skills are necessary to develop a good academic relationship with peers which eventually leads to better academic satisfaction [16]. On the other hand, students with higher intrapersonal intelligence have a better ability to understand how they feel, what emotions they have, and the changes that happen to them both physically and psychologically. This process is also called self-awareness which is very important for academic success [3]. It was formerly believed that IQ was sufficient to determine an individual’s aptitude. However, it was discovered that IQ alone is not enough to determine academic success, and instead, research has suggested that emotional skills are vital for practical life and yield positive results [17]. It was found that EI was a more accurate predictor of advancement than IQ [18].

Stress management is critical for academic and professional success. According to [19], developing emotional abilities aided students in effectively coping with stress and maintaining a healthy mental condition. Scholars have grown more interested in the frequency of academic stress among university students over the past several years. It has long been recognised that dental education is strongly connected with stressors [20]. Generally dental institutions have an inflexible academic atmosphere that places a premium on competitiveness over cooperation among students. Dental students have been subjected to stressful stimuli such as a strict and constrained study schedules, dealing with uncooperative patients, delicate methods, advanced work, and, most crucially, less personal time. Dental students must exhibit current knowledge to progress through a rigorous program [21]. Of final-year dentistry students, 67 % show signs of stress and pathological anxiety. Some 22% of first-year dentistry students have been diagnosed with persistent depression and emotional weariness [22]. A student’s expert viability may be harmed if stress impairs his or her ability to pay attention and concentrate, reduce his or her fundamental leadership abilities, and weaken his or her capacity to establish trusting connections with patients and colleagues. Student health and well-being might be jeopardized if the dental environment causes stress [23]. If stress is on-going, students might not be able to work, might have a hard time interacting with their patients, and might be depersonalized, which means they will be psychologically distant from other people. Students who are under a great deal of stress are more likely to fail academically and have fewer career and lifestyle possibilities [24,25]. Students who are stressed out in university due to academic pressure and personal situations may also be stressed out as dentists [26].

Here, EI has been found to be beneficial in coping with emotional and stressful situations [27]. Greater EI has been linked to less stress and improved managing abilities among medical students and other healthcare professionals [27,28]. Much research has demonstrated that those with EI skills provided superior consultations, were more empathic toward patients, communicated more effectively, and had superior therapeutic results [29,30]. Greater EI has been linked to less stress and improved managing abilities among medical students and other healthcare professionals [31].

This alludes to the possibility that EI is a critical factor in the development of professional and competent dentists. However, it is as yet unknown to what degree EI can be used to assess behavioral complexity, stress management, and academic achievement of dental students. However, there is little worldwide research on the influence of emotional intelligence on academic achievement and stress management among dentistry students. This may be because it is a unique concept that is gaining traction among health education researchers. As a result, it is vital to conduct a systematic and scientific assessment of the available data to understand how emotional intelligence is related to academic achievement and stress. The goal of this scoping review is to assess the impact of EI on personal and academic progress as well as stress management among dental students.

## 2. Methodology

### 2.1. Search Strategy

A primary evaluation of current literature was carried out to support the rationale for this systemic review following the guidelines of the Centre for Reviews and Dissemination (CRD) [32]. Several academic databases were scanned to detect corresponding primary studies. Academic resources such as Pubmed, Pubmed Central, EMBASE, Web of Science, EBSCOHost and ARU E-library were explored for this SR. Cochrane and PROSPERO were also scrutinized to ascertain if a current SR on the same topic existed. Several reviews were found evaluating the relationship between EI of medical, nursing undergraduate students and their academic performance during their undergraduate years. However, no scoping reviews were reported regarding EI of dental undergraduate students and academic performance.

**Figure 1:**
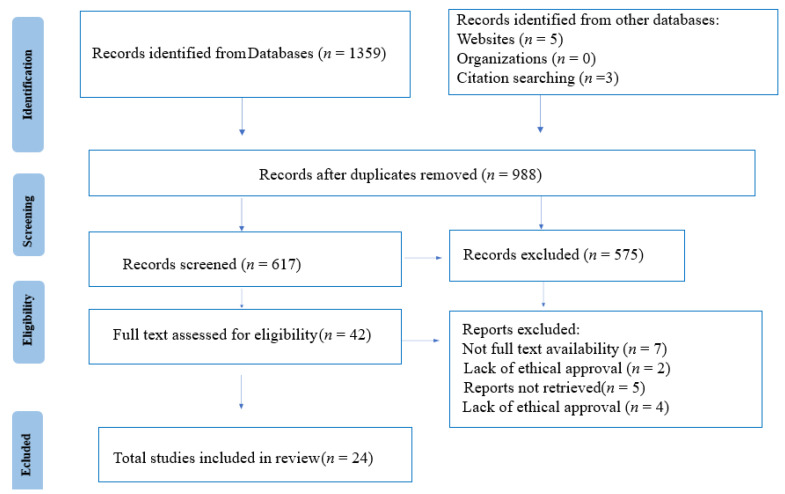
PRISMA Flowchart.

Following the Preferred Reporting Items for Systematic Reviews and Meta-Analyses (PRISMA), as shown in Figure 1 a detailed screening of the existing studies was performed to detect corresponding articles. Flowcharts were prepared from “Methodology for JBI Scoping Reviews” and the review was not registered [33]. Screenings of literature were not limited to region and were conducted across many databases to avoid missing relevant research and reduce bias. PubMed, JSTOR, Ovid MEDLINE, Ovid EMBASE, EBSCO CINAHL Plus, and BioMed Central were all checked. The keywords were identified with PICO search strategy tools owing to their acceptability as shown in Table 1 [34].

Boolean operator conjunctions were applied to yield more concentrated outcomes, and the MeSH browser was used to index articles. The search comprised the words “emotional intelligence” AND “student” AND “dental” AND “performance OR skills OR competence”. For assessing perceived stress, “emotional intelligence” AND “student” AND “dental” AND “stress factors” was used as shown in Table 2. Different synonyms were applied with OR to provide broader results.

The lists of search terms are given below—

**Table 2:** Search Items.

Emotional Intelligence	Dental	Academic Performance	Stress
Emotional intelligence (MeSH)	Dental education (keyword)	Academic performances	Stress disorders
Intelligence	Students	Academic test performances	Stress, psychological
Emotional intelligence	dental (MeSH)	Academic test scores	Dental stress analysis
Emotional social intelligence	Schools dental (MeSH)	Educational test performance	Occupational stress
Social intelligence		Educational test scores	

Search criteria were set to narrow the range. Only peer-reviewed full text publications in English were included. Moreover, the reference lists of relevant papers obtained from the database search were scanned to find additional articles.

The database search as shown in Table 3 yielded 1359 articles and three were extracted through reference harvesting.

### 2.2. Study Selection

To minimize duplication bias, the duplicated papers were manually checked and removed using RefWorks software before the inclusion and exclusion criteria interface. A total of 991 papers were detected after eliminating the redundant articles, followed by the literature search. Two researchers were involved in the study selection and screening process. Since the research objective does not contain either negative and pos itive outcomes, all published and unpublished studies are representatives of this project. Google Scholar was used to find publications or conference abstracts were not listed. Overall, five relevant papers were retrieved but omitted as they are not peer-reviewed. This search includes peer-reviewed publications as they provide better quality research, limiting bias.

### 2.3. Inclusion Criteria and Exclusion Criteria

Initially, the papers obtained from the search with imposing limitations were analyzed for study purposes. Secondly, headings and abstracts were screened to obtain relevant documents. The screening process included both qualitative and quantitative studies. The impact of EI among dental students was not addressed in some studies, therefore the “PI” and “O” inclusion and exclusion criteria were adopted. Fourthly, the search yielded 42 related papers to EI and later reviewed, focusing on the relationship between EI and academic performance and stress. Articles that presented ambiguous or limited information about EI and/or stress, academic performance among dental undergraduate students were discarded. Following the criteria as shown in Table 4, 31 papers were selected for the critical appraisal.

### 2.4. Data Extraction

A data abstraction table was created that incorporated the details about the authors, publication year, research methodology, population, career preference, stage of graduation; EI measurement tools and the main outcome related to academic performance and stress. Microsoft Excel was used to retrieve the data. The data comprised the article’s intext reference.

### 2.5. Analysis

As the scoping review incorporates data from both qualitative and quantitative research, a meta-analysis could not be performed. The data collected from the listed papers was organized and analyzed using Microsoft Excel. Following that, a textual narrative synthesis was performed. The term ‘narrative synthesis’ refers to a method for synthesizing and summarizing data from much research that highly depends on the use of words and literature to describe the synthesis’ findings.

### 2.6. Critical Appraisal

The critical appraisal assessed 31 papers according to the scientific strengths and flaws of studies and the reliability with the bias prevalence as shown in Table 5. The articles were reviewed utilizing respective types of evaluation methods such as qualitative studies were assessed using the Critical Appraisal Skills Program (CASP) as shown in Table S1 [34].

Cross-sectional studies were evaluated applying the Appraisal tool for Cross-Sectional Studies (AXIS), particularly for this type of study design as shown in Table S2 [35]. This assessment included an ethical approval to enhance the legal and empirical integrity of this study, avoiding articles containing ethical considerations.

The review provided 24 papers followed by critical and ethical evaluation. Seven studies were eliminated from this scoping due to a lack of ethical issues, low internal reliability of the study, which impacts the credibility of the conclusions.

**Table 5:** Data extraction table (characteristics of the 24 papers included in the review and summary of their findings).

Reference	Aim	Country	Sample	Scale	Study Design	Findings	Limitation
[36]	EI and stress coping in dental undergraduates	UK	213	EI Scale by Schutte	Qualitative	Students with a high EI score had a stronger capacity for reflection and assessment, as well as social and interpersonal skills, management, and multitasking. Students with a low EI were more inclined to take part in unhealthy lifestyles or activities.	The research made no attempt to assess the results of pathological anxiety. Apparent lack of quantitative and statistical analysis
[37]	EI of dental students and patient satisfaction	Iran	123	Bar-On Standardized Emotional Quotient Inventory (EQI)	Cross sectional	There may be a correlation between the EI score of dental students and their patient satisfaction. Female students might bene fit from stress management training.	There might be some overlap between core personalities and the EI component. The conclusion contradicts many other pertinent studies.
[38]	EI level among postgraduate pedodontics students	India	240	Goleman’s model of EI	Cross sectional	Both male and female pedo dontists in India had high EI scores Males had higher scores for dimensions of self-awareness, social awareness, and social skills.	The study group EI was assessed on a self-report scale They are prone to response biases.
[39]	Relationship between self-esteem, emotional intelligence, and empathy	Trinidad and Tobago	460	Jefferson Scale of Physician Empathy The Trait Meta Mood Scale—perceived emotional intelligence Rosenberg Self-Esteem Inventory	Cross sectional	A minor positive correlation exists between emotional intelligence and self-esteem, as well as between empathy and self-esteem. Male students performed much better on emotional intelligence tests. Those who identified as Indian showed somewhat greater self-esteem than those who classified as mixed/other.	Self-report instruments were utilized, which are prone to social desirability bias.
[40]	EI level among dental interns	Egypt	267	Genos EI self- assessment model	Cross sectional	Most dental interns had excellent EI ratings Female individuals had considerably lower EI values than male ones.	Susceptible to response biases because of self-report
[41]	EI and clinical interview performance of dental students	New Zealand	116	Social skills inventory (SSI) Communication skills marking sched ule (CSMS)	Cross sectional	Female students have more global social skills abilities and were more expressive and sensitive emotionally than male students, although males possessed greater emotional control. Students who speak English as a first language also performed better on all tests.	The course has a gender inequality, with a higher percentage of females than males. The interview assessments were conducted by teachers rather than anonymous reviewers.
[42]	The relationship between socio- demographic factors and EI and academic success in dental, clinical and preclinical courses	Pakistan	113	San Diego City College MESA Program from a model by Paul Mohapel	Cross sectional	EI is crucial for academic success in clinical courses. EI was somewhat higher in females than in males. Students who had siblings had considerably higher EI scores.	A greater proportion of female participants than male participants. The sample size for several socio-demographic variables was insufficient to generalize the findings.
[43]	EI and academic success	India	160	Emotional intelligence scale (situational)	Cross sectional	There is a considerable link between emotional intelligence and academic achievement.	Prone to response biases due to self-report. High number of female participants.
[44]	EI score and performance of dental undergraduates	Japan	129	Mayer–Salovey–Caruso Emotional Intelligence Test	Cross sectional	Female students had much higher EI scores than males. The EI score was greater in the group of high-grade academic achievers than in the group of low-grade academic performers.	EI tests were self- assessed by students. Cohorts were limited to students enrolled at a single dental university.
[45]	EI and empathy among medical and dental students	Pakistan	2170	Schutte Emotional Intelligence Scale Davis’ Interpersonal Re activity Index (IRI)	Cross sectional	Emotional intelligence was shown to vary significantly across medical and dentistry students from public and private institutions. When compared to dental students from public institutions, private-sector students demonstrated considerably greater levels of empathy. Females had considerably greater levels of emotional intelligence and empathy. Significantly more empathy and emotional intelligence in preclinical dentistry students compared to clinical dental students.	Prone to response biases due to self-report. Cohort study should be conducted.
[46]	Association between dental students’ EI and academic performance	India	200	EQSAC	Cross sectional	EI was shown to be significantly linked with academic success. Females had a considerably higher EI than males.	There was an uneven distribution of men and females in the sample. Biases such as social desirability, central tendency, and acquiescence may exist.
[47]	EI and subjective wellness among dental students	UK	218	Trait Emotional Intelligence Questionnaire-(TEIQue-SF) Rosenberg Self-Esteem scale	Cross sectional	EI was associated with subjective well-being and life satisfaction. Improved EI may lead to better life quality.	Participants may have evaluated themselves more positively. Casual self-evaluation.
[48]	EI and self-perception among medical and dental students	Pakistan	1172	Schutte Emotional Intelligence Scale (SEiS)	Cross sectional	There was a weak correlation between emotional intelligence and self-perception. Male and female students scored equally well on tests of emotional intelligence.	The sample was drawn from a single area. There is a possibility of response bias.
[49]	Impact of EI training in a communication and ethics	USA	120	EQSAC	Cross sectional	There were significant positive associations between EI levels and students’ perceptions of the relevance of EI. Integrating EI training into dentistry curriculum may have a beneficial effect.	Information was gathered from a cohort of dental students at a single institution. It is possible that self-reported information obtained via surveys was implicitly skewed.
[50]	Association between EI and academic performance	USA	63	EQSAC	Cross sectional	EI subgroup scores and clinical GPA were shown to be significantly linked. It was observed that self-control, motivation, and self-confidence are all determinants of total academic achievement. Clinical performance was shown to be predicted by social competence, empathy, and motivation.	The sample was mostly female, with just one male participant. This study did not examine personality characteristics or other elements associated with EI.
[51]	Relationship between EI and job satisfaction	Malaysia	581	Schutte Emotional Intelligence Scale	Cross sectional	EI was statistically substantially linked with JS in the following domains: patient connections, peer support, professional growth, quality of care, and staff support. Neither EI nor JS was statistically substantially connected with income.	The JS questionnaire used in our study was designed from a western setting and may not be appropriate for use by local dentists.
[52]	Emotional intelligence and perceived stress	England, Romania, South Africa, Australia, USA, Greece, Malaysia	741	Schutte Emotional Intelligence Scale	Cross sectional	EI and PS are inversely connected. Significant disparities in EI and PS scores across nations have been identified. When compared to their contemporaries, females, younger students, those without a prior higher education background, and those dissatisfied with their choice to pursue dentistry were more likely to experience PS.	This study emphasizes some of the personal aspects that accompanied dental school applicants, additional criteria were not analyzed.
[53]	Emotional intelligence among dental undergraduate students	India	186	EQSAC	Cross sectional	Only 11.55% students had good EI, and majority have moderate EI.	Self-reported scale, the validity of the data can be questioned. The results of the study are prone to response bias.
[54]	Relationship between EI and depression	Pakistan	200	Baron EQI Beck Depression Inventory	Cross sectional	Student depression has a significant negative moderate as sociation with emotional intelligence. The Emotional Intelligence Quotient of male and female medical and dentistry students is not significantly different.	Self-reported study. The study was conducted in a limited area with small sample size.
[55]	Association between emotional intelligence and perceived stress	Malaysia	234	Schutte Emotional Intelligence Scale	Cross sectional	EI ratings were much higher in females than in males, as well as in students who selected dentistry primarily on their own interests rather than on the encouragement of others. A negative association between EI and PSS-10 scores that is statistically significant.	In addition to EI, additional variables may have contributed to PSS results that were not explored.
[56]	Self-compassion and emotional intelligence of engineering and dental college students	India	60	Emotional Intelligence Scale by Schutte The Self Compassion Scale (SCS)	Cross sectional	Engineering students have a higher EQ than dentistry students. There is no statistically significant difference in self-compassion and emotional intelligence between females on the Dental and Engineering courses.	Sample sizes were not large enough. There may be response bias.
[57]	Relationship between emotional intelligence and academic satisfaction	Iran	80	EQI	Cross sectional	There is a positive association between EI and economic conditions, social status and economic conditions and interest of education. Positive relation between these EI and academic satisfaction.	Self-reported Study. There may be a chance of selection and response bias.
[58]	Relationship between EI and dental student clinical performance	USA	100	ECI-U Cluster ratings	Cross sectional	Student EI scores were shown to have a statistically significant impact on patient satisfaction. Male students fared better on stress regulation, overall mood, and intrapersonal measures than female students. Students who were married had better scores on adaptability.	There is a possibility of response bias. Performance is assessed using a single field and a constrained process.
[59]	Perceived stress among dental students	Western Cape	411	Maslach Burn- out Inventory (MBI)	Cross sectional	The level of stress rose during the school years. Students’ academic and professional development may be harmed by work stress. People who are older are more likely to have emotional exhaustion.	Findings were based on a single-centre, cross-sectional study comparing students from different years in the program. These results were obtained from a single School of Dentistry.

## 3. Results

### 3.1. Characteristics of Studies

The analysis comprised 24 studies from 15 countries. Fourteen studies were undertaken in Asian countries Iran, India, Pakistan, Malaysia, Japan. Five studies were conducted in North America and Caribbean countries USA, Western Cape, Trinidad, and Tobago. Two studies were performed in UK and one in New Zealand. Furthermore, one multinational collaborative studies in England, Romania, South Africa, Australia, USA, Greece, Malaysia. Five studies explored the relationship between emotional intelligence and academic performance among dental students. Five studies evaluated whether emotional intelligence helped dental students to cope with perceived stress. Six studies examined how emotional intelligence can assist dental students to achieve personal and job satisfaction. The remaining studies focused on consultation skills, patient satisfaction, empathy, and communication management. Some studies also demonstrated the difference between gender and EI score which varies in different countries.

### 3.2. Design of Studies

In one qualitative study, dental students were surveyed in detail and data was gathered via interviews. The remaining studies were cross-sectional studies that used questionnaire, in-person interview, or audio-computer aided interview to get relevant data.

### 3.3. Source of Information

The information retrieved from the studies was self-reported by the individuals who took part in the surveys. The studies comprised ages, socioeconomic classes, and year group. Some of the samples shared their own personal experiences with EI, while others responded to performance tests.

### 3.4. EI Assessment Methods

There are several theories regarding EI that use their own evaluation instruments, which usually involve performance or self-report activities, depending on what the theory considers is the best way to measure EI. Eighteen of the publications are based on Mayer and Salovey’s ability model (MSCEIT, TEIQue-SF, EQSAC, EQI, SSEIT, and C-EIS-R), one on a mixed method Bar-On model (EQ-i), one on Goleman’s mixed model (EIT), one on Genos EI self-assessment model. To evaluate the academic and clinical performance, the coping with stress, empathy, communication ability Social Skills Inventory (SSI), Communication Skills Marking Schedule (CSMS), Jefferson Scale of Physician Empathy, Rosenberg Self-Esteem Inventory, Maslach Burnout Inventory (MBI) were adopted.

### 3.5. EI and Academic Performance

Six papers examined the association between EI and the overall academic performance of dental students. Data from different dental institutions in Pakistan were collected to ascertain the effect of EI on academic performance in preclinical and clinical dental graduate courses [41]. They discovered that the development of EI is influenced by familial and societal factors. In clinical courses, EI is essential for higher academic achievement. Similar findings were observed in a Japanese study. There was a greater disparity in EI scores between the two groups of students based on their academic performance [43]. Three studies in India [42,45,53] demonstrated that EI and AP are connected, especially in clinical courses. It was found that there was no significant relationship between EI and academic scores among first and second year students. However, in the third and fourth years, the academic result is proportionate to EI score [42]. It was also noticed EI was linked to a wide range of parameters, including gender, sleep, staying with family, meeting friends, physical activity, leisure activities, and profession choice [45]. Moreover, a positive correlation of academic performance is associated with those domains. Final test performance represents constant evaluation of technical and interpersonal skills and professional attributes associated with EI. It was observed that only 11.55% of students obtained a high EI score and the majority of the students had average or poor EI [52]. As a result, dental students are lagging in academic competence alongside their interpersonal skills. Similar results were noted among dental hygienist students in USA [49]. Most of the students had moderate EI scores. Successful dental hygienists often possess not just a high level of intellect as seen by their GPAs, but also the ability to communicate effectively, with a talent for empathizing with and communicating with others effectively. Overall, academic performance was favorably connected with EI scores [49]. In Iran, the relationship between academic satisfaction and EI was evaluated among dental and paramedic students. The results indicated that academic satisfaction was more prevalent among dental students. EI was noticeably associated with economic condition, social status, and personal interests [56]. They indicated the positive relationship between these factors and academic satisfaction.

### 3.6. EI and Coping with Stress and Depression

A multinational collaborative research comprising nine dental schools from seven countries examined the relationship between perceived stress and gender, age, educational credentials, work performance, and EI [52]. An inverse association between EI and PS was identified in each country. It implies that students with better EI are better equipped to deal with the stressful demands of their training, bolstering the need for EI enhancement training. This transnational study established a negative relationship between EI and PS, regardless of students’ academic level or contentment with their profession choice [52]. Depression and emotional intelligence quotient are shown to be adversely associated [53], with a significant adverse association between students’ EI and PSS-10 levels [54]. Additionally, they observed that fourth-year students with the highest PS scores also had the lowest EI scores and students who selected dentistry for personal reasons had considerably lower PSS-10 results than those persuaded by others. This research emphasizes the importance of EI as a factor of PS. Stress levels were found to climb steadily throughout the academic years and peaked in the fourth year [58]. Fourth-year students tend to be the most vulnerable since they fulfil emotional adaptability requirements consistent with burnout. The authors suggested a substantial correlation between burnout syndrome prevalence and stress management. Students with higher EI are less prone to burnout and emotional exhaustion. According to the findings of a qualitative research [36], stress is perceived emotionally. Students with a high EI score had a more substantial capacity for contemplation and assessment and social and interpersonal skills, organization, and multitasking. Students with a high EI reported being more organized in their activities and managing their working time confidently, while students with a low EI reported being hesitant to structure their time [36].

### 3.7. EI and Clinical Competence

A study [38] among pedodontics students revealed that students who have higher EI were more likely to develop reflection and assessment, interpersonal and organizational skills, as well as time management abilities, all of which are necessary for a successful clinical career. Dental clinical work is stressful. Students are expected to develop a range of cognitive, psychomotor, and interpersonal abilities. EI abilities are found critical for a clinical course [41]. An inherent connection between cognitive capacity and didactic competence, as well as between emotional intelligence and clinical efficiency was also revealed [57]. It was also found that all these participants had the minimum level of information essential for clinical efficacy and that what distinguished them clinically was mostly due to variations in their emotional intelligence abilities, specifically, their self-management competencies. Students can enhance their EI abilities across several contexts of consultation skills, including receptive listening and adaptiveness to the patients’ needs and feelings, scooping up on the clients’ verbal and gestural behavior, displaying information about a patient, empathy, sensitivity, and denial of negative judgments [56]. Dental students’ clinical performance and their EI score may be linked [37], therefore teaching students about EI aspects may help them acquire the interpersonal skills they will need to be successful in their careers as dental practitioners.

### 3.8. EI and Empathy, Perception, Self-Esteem, and Job Satisfaction

Emotional intelligence, self-esteem, and empathy all seem to be interconnected, but it is hard to say exactly how they work together. In [39], the authors noted that empathy and emotional intelligence have a strong association with self-esteem, though the relation is relatively smaller among first-year students. These students have not had much experience with their fields, especially in a clinical setting, so it is likely that they will not have a lot of interpersonal communication and societal assimilation in their disciplines. Similar attempts were taken by [44] to compare EI levels between government and private dental college students. Dental students who went to private institutions showed a lot more empathy than students who went to government universities. Additionally, they discovered that first-year pre-clinical dentistry students scored much higher on empathy than the following years’ students which is totally contradictory to [39] findings. Saddki et al. indicated substantial correlation between emotional intelligence on the surface, core self-evaluations, and subjective well-being among dentistry students [46]. On the other hand, Pakistani dental students’ poor coordination between emotional intelligence and self-perception was detected [47]. In Malaysia, EI is linked to career satisfaction in several facets of a dentist’s professional life. These factors include patient connections, peer, career advancement, clinical outcomes, and employee relationships. Additionally, they noted that sociodemographic and occupational factors were not statistically significantly related to EI or job satisfaction [50]. Dental students who received EI training as part of a communication and ethics course reported higher levels of EI [48]. Dental students generally acknowledged their enhanced self awareness of EI, focused on the personal potential for growth, and saw the connection between EI and clinical management in the clinical setting [48]. There was a strong correlation between emotional intelligence and empathy among dental students [55].

### 3.9. EI and Gender

Males had greater EI scores in a few studies [37,39,40,41], but the opposite claim also being noticed that women had higher EI scores [42,45,46,47,48,49,50]. However, the rest of the studies did not discover a statistically significant difference in EI between male and female. The gendered EI reports from various geographical regions are somewhat varied. It is also worth noting that males scored better on the aspects of self-awareness, social awareness, and social skills. This might suggest that they have slightly higher EI capacities than women. Male students scored higher on the self-awareness component than female students, indicating that they were more conscious of their internal states and personal skills. Females have a greater EI due to more empathy, a distinction in socializing processes, and a bigger emotional processing region in the mind. Female students were more emotionally expressive and sensitive than male classmates. Females, on the other hand, experienced more stress than men. They are more prone to seek assistance for psychological difficulties and to use mental health services [54].

## 4. Discussion

Dental training entails patient engagement and communication, so it is reasonable to assume that students with high EI scores will produce more positive clinical outcomes and will be regarded more favorably by their clients. The review showed that EI plays a bigger role in the educational excellence of dental students in their clinical years. This is particularly true for their interpersonal competence, as proven by analysis demonstrating a substantial positive association between communication skills and EI or its subscales. Additionally, the association between EI and holistic academic achievement throughout the clinical years supports an indirect function for EI in the learning of communication skills. Dental courses are assessed by written tasks, tests, and clinical evaluation. This involves the acquisition of extra interpersonal skills such as empathy, effective listening, and clear communication to reduce patient stress and handle patient demands. These abilities are critical for enhancing health outcomes for patients, patient contentment, and medication compliance. Throughout the clinical sessions, students must demonstrate cooperation and management planning abilities to meet all clinical obligations. These extra competencies will benefit the practical training and performance during clinical evaluations. As a result, these students with a higher EI are projected to be more adept at seeing, comprehending, and managing their own and others’ moods, and hence do well in clinical coursework [59]. It is possible that those who have a higher level of certain of the competences in relationship management may be more inclined to create study groups, which might lead to greater academic performance. Students with excellent grades may use emotions to better their cognition, grasp the complexities of emotional meanings or circumstances, regulate their own and other people’s emotions, and use emotional information for planning and consciousness. These findings are also supported by [60], where the positive impact of emotional intelligence on academic performance among medical students were systematically demonstrated. Effective communication involves a complicated process including the perception of emotions, self-control, and the use of emotions to assist in future performance [61]. Patient dissatisfaction in dental practice is most often caused by the dentist’s poor communication skills, notwithstanding his or her good academic knowledge and technical abilities [62,63,64]. On the other hand, a dentist with high emotional intelligence can successfully communicate with worried patients and persuade them toward positive lifestyles. This lessens patient fear and increases compliance with dental recommendations, resulting in increased client and dentist satisfaction [65]. EI may be a factor in how well intrapersonal and interpersonal relationships work and how well dentists do their jobs, and it may also predict whether a dentist will be successful in their career. Dental students with higher EI scores are often more adept at coping with academic and other forms of stress. Stress reduction results in increased academic achievement and patient satisfaction. Students who can manage their emotions, avoid overreacting to negative emotions, and persevere in a tough setting are less likely to experience psychological discomfort. It was found that students with a higher EI were more inclined to develop contemplative and evaluation skills, interpersonal and intrapersonal, organizational, and leadership qualities, all of which are necessary for job progress.

Studies in India, Pakistan, Iran, Egypt, and Trinidad revealed that males possessed greater EI scores, while studies in UK, USA, Japan, Malaysia, Australia, New Zealand, Greece, and Romania found that females had higher EI scores. Even a few studies in India and Pakistan supported female dominance. However, other studies in health care found no significant difference in EI between males and females [66]. This may reflect the many cultural distinctions across communities, with female dentists in wealthy nations having a better ability to express their emotions and behave freely with their patients than in underdeveloped ones. Multiple explanations have been advanced to explain gender disparities in EI scores. According to researchers, biological differences, and disparities in early childhood socialization within same-sex playgroups, including compliance with culturally dictated gender role expectations, may be the key factors [67]. The overall findings of this research indicate that there is no substantial gender variation in dental students’ emotional intelligence and empathy.

## 5. Limitations

The study evaluated 24 research publications that focused on dental students. It was difficult to generate a consistent conclusion since each study utilized a different EI measuring method and very few included response rates. Moreover, there was high heterogeneity across the different studies. While investigating the connection between EI and objective techniques of evaluation, it was discovered that not much research had expanded on appraisal characteristics such as domain assessment, types of questions, weightage, and frequency of exams. Finally, the evaluation has solely considered college grades, such as end-of-year marks, GPA, Clinical Practice Examination scores, or articulation scores. Only traditional learning outcomes were assessed objectively using these instruments. Professionalism, ethical conduct, capacity to create a therapeutic connection with the patient, and patient or family satisfaction have all been connected to the professional success of a dentist. However, due to lack of information, these traits were not considered in this study. To overcome the issue, more research should be incorporated through a modification in search methodology. Additionally, it was challenging to combine all factors relevant to emotional intelligence in this review.

## 6. Conclusions

The review reveals that students with a higher EI may manage better with the stressful demands of their training, bolstering the case for including EI training in the curriculum of students of the health professions. Its inclusion in the dental curriculum may assist students in identifying their emotional characteristics and providing opportunities to teach them to a higher degree of competence in dealing with emotional intelligence, consequently increasing academic performance. Furthermore, this study bolsters the claim that emotional intelligence (EI) and the capacity to handle stress are essential traits of professional competence, and that EI should be used as a selection criterion for admission to dental education.

## Figures and Tables

**Table 1:** PICO search tool.

	PICO Search Tool
P—Population	Undergraduate and postgraduate dental students
I—Intervention	Emotional intelligence
C—Comparison	None
O—Outcome	Academic performance, stress factors

**Table 3:** Comprehensive database-analysis.

SL NO.	Database	Related Articles Found
1	PubMed	203
2	CINAHL Plus	17
3	Medline	31
4	JSTOR	726
5	EMBASE	12
6	BioMed Central	370
7	Reference harvesting	3

**Table 4:** Inclusion and exclusion criteria.

	Inclusion	Exclusion
Population (P)	Dental students (both undergraduate and postgraduate)	Students from other sectors, such as medical, engineering, business, or humanities, etc.
Intervention (I)	Emotional intelligence	Other factors such as IQ, background, economic solvency, etc.
Comparison (C)	Peer-reviewed studies published from 2004 to 2020	Studies before 2004 mixed method studies Grey literature
Outcome (O)	Academic performance and stress factors	Other than academic performance and stress factors

## Data Availability

Not applicable.

## Supplementary Materials

The following supporting information can be downloaded at: https://www.mdpi.com/article/10.3390/dj10040067/s1, Table S1: Critical appraisal for qualitative studies using the Critical Appraisal Skills Program (CASP) tool.; Table S2: Critical appraisal for cross-sectional studies using the Appraisal tool for Cross-Sectional Studies (AXIS).
