# Supplementary material for: Exploring the Association between Emotional Intelligence and Academic Performance and Stress Factors among Dental Students: A Scoping Review
Source: Dent J (Basel). 2022 Apr 7;10(4):67. doi: 10.3390/dj10040067 (PMC9026633; doi:10.3390/dj10040067)
Supplement: Supplementary file 1 [file dentistry-10-00067-s001.zip › dentistry-1602389-supplementary.pdf]

dentistry journal

MDPI

Supplementary Materials:

Table S1. Critical appraisal for qualitative studies using the Critical Appraisal Skills Program (CASP) tool.

**List of Included qualitative studies.**										
**Qualitative Studies: CASP Tool**	**Section A: Are the Results Valid?**						**Section B: What Are the Results?**			
Title	Was there a clear statement of the aims of the research?	Is a qualitative methodology appropriate?	Was the research design appropriate to address the aims of the Research?	Was the recruitment strategy appropriate to the aims of the research?	Was the data collected in a way that addressed the research issue?	Has the relationship between researcher and participants been adequately considered?	Have ethical issues have been taken into consideration?	Was the data analysis sufficiently rigorous?	Is there a clear statement of findings?	How valuable is the research?
Emotional intelligence and stress coping in dental undergraduates—a qualitative study	+	+	+	+	+	+	+	+	+	+
List of Excluded Qualitative Studies										
Qualitative Studies: CASP Tool	Section A: Are the Results Valid?						Section B: What Are the Results?			
Reference	Was there a clear statement of the aims of the research?	Is a qualitative methodology appropriate?	Was the research design appropriate to address the aims of the research?	Was the recruitment strategy appropriate to the aims of the research?	Was the data collected in a way that addressed the research issue?	Has the relationship between researcher and participants been adequately considered?	Have ethical issues have been taken into consideration?	Was the data analysis sufficiently rigorous?	Is there a clear statement of findings?	How valuable is the research?
The effect of Emotional Intelligence on Research Quality and Teaching skills of Faculty in University	+	+	+	−	−	+	+	−	−	−
Robinson, P.G. Perceptions of professionalism in dentistry—a qualitative study.	+	+	+	−	+	−	+	+	−	−

(+) = item adequately addressed, (−) = item not adequately addressed, (+/−) = item partially addressed.

Table S2. Critical appraisal for cross-sectional studies using the Appraisal tool for Cross-Sectional Studies (AXIS).

List of Included Cross-Sectional Studies											
**Cross-Sectional Studies CASP Tool**	Introduction					Method					
Title	Were the aims/objectives of the study clear?	Was the study design appropriate for the stated aim(s)?	Was the sample size justified?	Was the target/reference population clearly defined? (is it clear who the research was about?)	Was the sample frame taken from an appropriate population base so that it closely represented the target/reference population under investigation?	Was the selection process likely to select subjects/participants that were representative of the target/reference population under investigation?	Were measures undertaken to address and categorize non-responders?	Were the risk factor and outcome variables measured appropriate to the aims of the study?	Were the risk factor and outcome variables measured correctly using instruments/measurements that had been trialed, piloted or published previously	Is it clear what was used to determine statistical significance and/or precision estimates? (e.g., *p*-values, confidence intervals)	Were the methods (including statistical methods) sufficiently described to enable them to be repeated?
Emotional intelligence of dental students and patient satisfaction	+	+	+	+	+	+	+	+	+	+	+
Emotional intelligence among dental undergraduate students: an indispensable and ignored aspect in dentistry	+	+	+	+	+	+	+	+	+	+	+
Emotional intelligence and perceived stress in dental undergraduates: a multinational survey	+	+	+	+	+	+	+	+	+	+	+
Relationship between emotional intelligence and job satisfaction in newly qualified dentists	+	+	+	+	+	+	+	+	+	+	+
Impact of emotional intelligence training in a communication	+	+	+	+	+	+	+	+	+	+	+

and ethics course among second-year dental students											
Association between emotional intelligence and academic performance among dental hygiene students	+	+	+	+	+	+	+	+	+	+	+
Emotional intelligence and self-perception of medical and dental students of Peshawar–Pakistan	+	+	+	+	+	+	+	+	+	+	+
Do core self-evaluations and trait emotional intelligence predict subjective well-being in dental students?	+	+	+	+	+	+	+	+	+	+	+
Association between dental students' emotional intelligence and academic performance: A study at six dental colleges in India	+	+	+	+	+	+	+	+	+	+	+
Do we need to care: emotional intelligence and empathy of medical and dental students?	+	+	+	+	+	+	+	+	+	+	+
Emotional intelligence score and performance of dental undergraduates	+	+	+	+	+	+	+	+	+	+	+
Emotional intelligence and its correlation with academic performance of dental undergraduates.	+	+	+	+	+	+	+	+	+	+	+

Association of socio-demographic factors and emotional intelligence with academic performance in clinical and preclinical dental courses	+	+	+	+	+	+	+	+	+	+	+
Emotional intelligence and clinical interview performance of dental students	+	+	+	+	+	+	+	+	+	+	+
The relationship between self-esteem, emotional intelligence, and empathy among students from six health professional programs	+	+	+	+	+	+	+	+	+	+	+
Emotional intelligence among Alexandria University dental interns	+	+	+	+	+	+	+	+	+	+	+
Emotional intelligence of Pedodontics and Preventive Dentistry postgraduate students in India.	+	+	+	+	+	+	+	+	+	+	+
Association between emotional intelligence and perceived stress in undergraduate dental students.	+	+	+	+	+	+	+	+	+	+	+
The relationship between depression and emotional intelligence quotient among the medical and dental students of Nishtar College Multan.	+	+	+	+	+	+	+	+	+	+	+
Self-compassion and emotional	+	+	+	+	+	+	+	+	+	+	+

intelligence of engineering and dental college students											
Perceived stress among dental students at the University of the Western Cape	+	+	+	+	+	+	+	+	+	+	+
What is the relationship between emotional intelligence and dental student clinical performance?	+	+	+	+	+	+	+	+	+	+	+
Relationship between emotional intelligence and academic satisfaction in dental students and paramedic students.	+	+	+	+	+	+	+	+	+	+	+
List of Excluded Cross-Sectional Studies											
Cross-Sectional Studies CASP Tool	Introduction						Method				
Reference	Were the aims/objectives of the study clear?	Was the study design appropriate for the stated aim(s)?	Was the sample size justified?	Was thetarget/reference population clearly defined? (is it clear who the research was about?)	Was the sample frame taken from an appropriate population base so that it closely represented the target/reference population under investigation?	Was the selection process likely to select subjects/participants that were representative of the target/reference population under investigation?	Were measures undertaken to address and categorize non-responders?	Were the risk factor and outcome variables measured appropriate to the aims of the study?	Were the risk factor and outcome variables measured correctly using instruments/measurements that had been trialed, piloted or published previously?	Is it clear what was used to determine statistical significance and/or precision estimates? (e.g., *p*-values, confidence intervals)	Were the methods(including statistical methods) sufficiently described to enable them to be repeated?
The impact of maternal emotional intelligence and parenting style on child anxiety and behavior in the dental setting	+	+	–	–	–	+–	–	+	–	+	–
Factors affecting clinical dental hygienist	+	+–	+	–	–	+–	–	–	+	+	–

emotional intelligence on burnout											
Relationships between emotional intelligence, ego resilience, stress in clinical practice of dental hygiene students	+–	+	–	–	+	–	–	+	–	+	–
The relationship between emotional intelligence and academic achievement in the dental students of Babol medical university	–	+	–	–	+	+	+–	+	–	–	+
Empathy and emotional intelligence in dental practitioners of Bareilly City—a cross sectional Study.	+	–	–	+	–	+	+	–	–	+	–

List of Included Cross-Sectional Studies									
Cross-Sectional Studies CASP Tool	Results					Discussion		Others	
Reference	Were the basic data adequately described?	Does the response rate raise concerns about non-response bias?	If appropriate, was information about non-responders described?	Were the results internally consistent?	Were the results presented for all the analyses described in the methods?	Were the authors' discussions and conclusions justified by the results?	Were the limitations of the study discussed?	Were there any funding sources or conflicts of interest that may affect the authors' interpretation of the results?	Was ethical approval or consent of participants obtained?
Emotional intelligence of dental students and patient satisfaction	+	+	+	+	+	+	+	+	+
Emotional intelligence among dental undergraduate students: an indispensable and ignored aspect in dentistry	+	+	+	+	+	+	+	+	+
Emotional intelligence and perceived stress in dental undergraduates: a multinational survey	+	+	+	+	+	+	+	+	+
Relationship between emotional intelligence and job satisfaction in newly qualified dentists	+	+	+	+	+	+	+	+	+

Impact of emotional intelligence training in a communication and ethics course among second-year dental students	+	+	+	+	+	+	+	+	+
Association between emotional intelligence and academic performance among dental hygiene students	+	+	+	+	+	+	+	+	+
Emotional intelligence and self-perception of medical and dental students of Peshawar–Pakistan	+	+	+	+	+	+	+	+	+
Do core self-evaluations and trait emotional intelligence predict subjective well-being in dental students?	+	+	+	+	+	+	+	+	+
Association between dental students' emotional intelligence and academic performance: A study at six dental colleges in India	+	+	+	+	+	+	+	+	+
Do we need to care? emotional intelligence and empathy of medical and dental students	+	+	+	+	+	+	+	+	+
Emotional intelligence score and performance of dental undergraduates	+	+	+	+	+	+	+	+	+
Emotional intelligence and its correlation with academic performance of dental undergraduates.	+	+	+	+	+	+	+	+	+
Association of sociodemographic factors and emotional intelligence with academic performance in clinical and preclinical dental courses	+	+	+	+	+	+	+	+	+
Emotional intelligence and clinical interview performance of dental students	+	+	+	+	+	+	+	+	+
The relationship between self-esteem, emotional intelligence, and empathy among students from six health professional programs	+	+	+	+	+	+	+	+	+

Emotional intelligence among Alexandria University dental interns	+	+	+	+	+	+	+	+	+
Emotional intelligence of Pedodontics and Preventive Dentistry postgraduate students in India.	+	+	+	+	+	+	+	+	+
Association between emotional intelligence and perceived stress in undergraduate dental students.	+	+	+	+	+	+	+	+	+
The relationship between depression and emotional intelligence quotient among the medical and dental students of Nishtar College, Multan.	+	+	+	+	+	+	+	+	+
Self-compassion and emotional intelligence of engineering and dental college students	+	+	+	+	+	+	+	+	+
Perceived stress among dental students at the University of the Western Cape	+	+	+	+	+	+	+	+	+
What is the relationship between emotional intelligence and dental student clinical performance?	+	+	+	+	+	+	+	+	+
Relationship between emotional intelligence and academic satisfaction in dental students and paramedic students.	+	+	+	+	+	+	+	+	+
List of Excluded Cross-Sectional Studies									
Cross-Sectional Studies CASP Tool	Results					Discussion		Others	
Reference	Were the basic data adequately described?	Does the response rate raise concerns about non-response bias?	If appropriate, was information about non-responders described?	Were the results internally consistent?	Were the results presented for all the analyses described in the methods?	Were the authors' discussions and conclusions justified by the results	Were the limitations of the study discussed?	Were there any funding sources or conflicts of interest that may affect the authors' interpretationof the results?	Was ethical approval or consent of participants attained?
The impact of maternal emotional intelligence and parenting style on child anxiety and behavior in the dental setting	+	−	−	+	−	−	+	+	+

Factors affecting clinical dental hygienist emotional intelligence on burnout	+	+–	+	–	–	+	–	–	+
Relationships among emotional intelligence, ego resilience, stress in clinical practice of dental hygiene students	+–	+	–	–	+	–	–	+	+–
The relationship between emotional intelligence and academic achievement in the dental students of Babol Medical University	–	+	–	–	+	–	–	+	+
Empathy and emotional intelligence in dental practitioners of Bareilly City—a cross sectional study.	+	–	–	+	–	–	+	+	+

(+) = item adequately addressed, (−) = item not adequately addressed, (+/−) = item partially addressed.
